# Mitigation of the Adverse Impact of Copper, Nickel, and Zinc on Soil Microorganisms and Enzymes by Mineral Sorbents

**DOI:** 10.3390/ma15155198

**Published:** 2022-07-27

**Authors:** Jadwiga Wyszkowska, Agata Borowik, Magdalena Zaborowska, Jan Kucharski

**Affiliations:** Department of Soil Science and Microbiology, University of Warmia and Mazury in Olsztyn, Plac Łódzki 3, 10-727 Olsztyn, Poland; agata.borowik@uwm.edu.pl (A.B.); m.zaborowska@uwm.edu.pl (M.Z.); jan.kucharski@uwm.edu.pl (J.K.)

**Keywords:** heavy metals, soil microbiome, soil enzymes, sorbents, *Helianthus annuus* L.

## Abstract

Despite numerous studies on the influence of heavy metals on soil health, the search for effective, eco-friendly, and economically viable remediation substances is far from over. This encouraged us to carry out a study under strictly controlled conditions to test the effects of Cu^2+^, Ni^2+^, and Zn^2+^ added to soil in amounts of 150 mg·kg^−1^ d.m. of soil on the soil microbiome, on the activity of two oxidoreductases and five hydrolases, and on the growth and development of the sunflower *Helianthus annunus* L. The remediation substances were a molecular sieve, halloysite, sepiolite, expanded clay, zeolite, and biochar. It has been demonstrated that the most severe turbulences in the soil microbiome, its activity, and the growth of *Helianthus annunus* L. were caused by Ni^2+^, followed by Cu^2+^, and the mildest negative effect was produced by Zn^2+^. The adverse impact of heavy metals on the soil microbiome and its activity was alleviated by the applied sorbents. Their application also contributed to the increased biomass of plants, which is significant for the successful phytoextraction of these metals from soil. Irrespective of which property was analysed, sepiolite can be recommended for the remediation of soil polluted with Ni^2+^ and zeolite—for soil polluted with Cu^2+^ and Zn^2+^. Both sorbents mitigated to the highest degree disturbances caused by the tested metals in the soil environment.

## 1. Introduction

The rapid pace of urbanisation and industrial development in the past decades has led to the global pollution with heavy metals, including copper, nickel, and zinc [1]. This issue is considered to be a particularly serious environmental challenge, especially in terms of crops and soils, all over the world, mainly in Europe, Australia, and North America [2]. It has also given rise to the imperative to redefine and give attention to the already existing concept of soil health, defined by Doran and Zeisse [3] as “the capacity of soil to function as a vital living system, within ecosystem and land-use boundaries, to sustain plant and animal productivity, maintain or enhance water and air quality, and promote plant and animal health”. Any disturbance of the identified soil’s functions is worrying, particularly in light of the fact that there are already 400,000 ha of agricultural ecosystems in Denmark, Germany, Finland, and Spain, as well as 200,000 ha in Hungary and Slovakia and around 600,000 ha in the USA, that require remediation [4,5]. This is a consequence of the toxicity, bioaccumulation, and biomagnification of heavy metals in soils [6]. Social unrest is additionally fuelled by the statistics which show that around 3.4 million tons of copper and one million tons of nickel are released to soils worldwide every year [7]. There are also alarming reports on the scale of contamination of agricultural land with sewage sludge, the main source of heavy metals, including zinc, which translates into 20 million ha of land exposed to contamination with these xenobiotics [8]. However, the widespread use of copper, nickel, and zinc, which leads to their dissipation in the environment, is accompanied by a low social awareness of the risks that these metals create [9].

The widespread use of copper is propelled by its high thermal and electrical conductivity, as well as resistance to corrosion [10]. This is why it is broadly used in power generation and in the electronics industries, in the manufacture of machines and furniture, and in the minting of coins [11]. In China, copper is considered to be an important, strategic raw material, second only to crude oil. However, the processing of copper generates large amounts of waste [10]. Significantly, copper oxide nanoparticles (CuONP) are gaining more interest and increasingly often used in gas sensors, solar energy conversion devices, and photovoltaic installations, as well as in agriculture and medicine [12,13,14]. Long-term exposure to copper is linked to growth and development disorders, carcinogenesis, mental retardation [15], and Parkinson’s disease, induced by increasing aggregation and toxicity of α-synuclein [16]. In turn, nickel is the 22nd most common element in the Earth’s crust, thus being twice as abundant as copper [17]. However, anthropogenic activities such as discharge of wastewater from the electroplating industry, production of cadmium batteries, nickel steel, and iron alloys accelerate the release of this element to soil [18,19,20]. Other sources of nickel in the environment are combustion of fossil fuels, generation of electricity, mining, and the cement industry [21,22]. This heavy metal exacerbates symptoms of Crohn’s disease [23]. Exposure to nickel is also implicated in asthma, chronic obstructive pulmonary disease, pulmonary fibrosis, and neoplastic lesions [22]. Similarly, zinc induces public concerns dictated not only by the production, but also by the toxicity of this heavy metal to humans.

China is the global leader in the production of zinc, with the annual output of 5 × 10^9^ kg [24]. Apart from sewage sludge, other significant sources of zinc are chemical fertilisers, including phosphates, broadly applied in agricultural land [25], car batteries, explosives, and car fuels, but zinc is also used in aeronautics, biosensors, and semiconductors [26,27]. Over 300 enzymes have been identified in the human body that require zinc as an essential cofactor [28], yet excessive amounts of this metal manifest toxic effects. Nickel causes cardiovascular disorders and cancers [29]. It is also responsible for Alzheimer’s disease, Parkinson’s disease, and Huntington’s disease [30].

Measures taken to minimise threats posed by the contamination of soil with copper, nickel, and zinc are undeniably substantiated. Considering the limitations of conventional soil remediation techniques, new perspectives are opening up for the use of heavy-metal sorbents, owing to their low cost, availability, and ability to exchange ions and to be incapsulated in crystalline structures of reactive minerals [31,32]. The most recent studies have also demonstrated that biochar, an eco-friendly product of pyrolysis, alleviates the adverse effects of soil pollution with heavy metals [33] by increasing carbon sequestration in soil, as well as by improving soil fertility and the stability of soil aggregates [34]. The main structural component of biochar prepared from straw residues, crop residues, manure, cereals, and grasses is carbon [35]. Its concentration is 17–96%. Phosphorus is present in 0.27–48%, with potassium in 0.1–5.8% and nitrogen in 0.18–5.60%. Minor quantities of sulphur, hydrogen, oxygen, and other basic cations are also present [36]. The properties of biochar vary according to the pyrolysis conditions, i.e., temperature, retention/heating time, pressure, inert gas flow, and chemical additives [33]. The addition of phosphorus compounds, silicon compounds, or hydrogen peroxide before the pyrolysis of the raw material can significantly modify its surface, functional groups, and surface charges, thus improving the efficiency of biochar for removing heavy metals [37,38]. An important role is also attributed to hyperaccumulation of heavy metals that over 640 plant species, including 0.25 of angiosperms, are capable of [39]. Nevertheless, copper, nickel, and zinc accumulated in amounts above 100 µg metal·g^−1^ of plant disturb the plant’s morphological, physiological, and biochemical processes irrespective of a plant species [40]. Toxicity of heavy metals associated with the disruption of the cellular redox system contributes to the formation of ROS (reactive oxygen species) and is responsible for peroxidation of lipids [41,42]. Other undesirable effects of the pressure of copper, nickel, and zinc include disorders in the processes of photosynthesis, evapotranspiration, and nutrient absorption [43]. 

Sophisticated mechanisms of chemical communication between microorganisms and plants are associated with plants releasing flavonoids, siderophores, and metallothioneins, which relieve the stress induced by the pressure of heavy metals [44]. Likewise, secondary metabolites induced by bacteria, e.g., acyl homoserine lacton (acyl HSL), are autoinducers directly activating gene pathways engaged in the regulation of tolerance to heavy metals [45]. The selective pressure of these xenobiotics exerted on the soil microbiome is also related to the adsorption of heavy metals by sulphate, amine, and carboxyl functional groups on the surface of microorganisms, and their absorption capacity for metal ions ranges from 1 mg·g^−1^ to 500 mg·g^−1^ bacteria [46]. Knowledge of the effect of heavy metals on both soil microorganisms and the activity of soil enzymes, which are considered to be early indicators of changes in the intensity of biological processes, is fundamental to an integrated evaluation of the soil condition [47,48,49]. 

Considering the phytoremediation potential of the sunflower (*Helianthus annunus* L.) [50] and the adsorption capability of reactive metals towards heavy-metal ions [32], a holistic study was conducted in order to determine the response of soil microorganisms and enzymes to soil contamination with Cu^2+^, Ni^2+^, and Zn^2+^. The suitability of a molecular sieve, halloysite, sepiolite, expanded clay, zeolite, and biochar was evaluated in terms of the remediation of soil cropped with the sunflower and exposed to the pressure of these xenobiotics. 

## 2. Materials and Methods

### 2.1. Characteristics of Soil and Sorbents, and the Preparation of Metal Solutions

#### 2.1.1. Soil

The soil used in the experiment was sampled from the top horizon (0–0.20 m) of an agriculturally used field located in north-eastern Poland (latitude 53.7167° N, longitude 20.4167° E). Soil was sifted through a sieve with the mesh size of 0.5 cm. Next, the basic physicochemical properties of soil were determined: pH, sum of exchangeable base cations, hydrolytic acidity, cation exchange capacity, alkaline cation saturation, and the content of organic carbon, total nitrogen, copper, nickel and zinc. The basic soil characteristics are given in Table 1.

#### 2.1.2. Sorbents

Six different loose sorbents were tested: a molecular sieve, halloysite, sepiolite, expanded clay, zeolite Bio.Zoe.S.01, and biochar.

The molecular sieve was a crystalline aluminosilicate with 0.3 mm micropores. This is a product sold under the trade name Silosiv A3 by the company Grace, Enriching Lives, Everywhere (the USA). The Sylosiv A3 molecular sieve is a zeolite with a three-dimensional pore system with pH_KCl_—8.5. Halloysite (Al_2_Si_2_O_5_(OH)_4_) is a clay mineral characterised by large proper surface, porosity, and ion exchangeability. It is used for making mineral sorbents. The halloysite used in this experiment was obtained from the company Intermark (Gliwice, Poland). Its chemical composition is as follows: SiO_2_—40 ± 1%, Fe_2_O_3_/FeO—8 ± 1%, TiO_2_ —2 ± 1%, Al_2_O_3_ —33 ± 1%, MgO—0.5 ± 0.1%, CaO—1.3 ± 0.2%, Na_2_O—0.1%, and K_2_O—0.1%, with pH_KCl_—5.9. Sepiolite (Mg_4_[Si_6_O_15_(OH)_2_]6H_2_O) is hydrated magnesium silicate with sorption properties. The sepiolite tested in the experiment was made by the company Sepiolsa Minersa Group (Spain) and sold under the name Sepiolite 60/100. Expanded clay is light ceramic aggregate produced by burning clay minerals and clays at a temperature of 1200 °C. It is a highly porous material. According to the manufacturer’s data, 85% sepiolite consists of particles with a size of 75 to 710 μm, with pH_KCl_—7.1. In our study, it was purchased from the company Garden Guru (Piła, Poland). Zeolite Bio.Zeo.S.01 is a sorbent which is a natural zeolite. It was made by the company BioDrain (Rzeszów, Poland). The chemical composition is as follows: SiO_2_—70.6, Al_2_O_3_—12.32, Fe_2_O_3_—1.48, TiO_2_—0.71, MnO_2_—0.02, CaO—3.42, MgO—0.96, K_2_O—2.83, and Na_2_O—0.68 (% of oxides). Biochar with pH_KCl_—8.0 is a substance made from organic matter through thermal conversion in anaerobic atmosphere. The biochar used in our experiment was made by the company Fluid (Poland). It contains more than 77% stable C, 17% volatile matter, less than 6% ash, and less than 0.01% chlorides, sulphur, and mercury. Complete specification of the molecular sieve, halloysite, sepiolite, and biochar was presented in our earlier paper [51], whereas zeolite Bio.Zeo.S.01 was characterised in the article by Boros-Lajszner et al. [52].

#### 2.1.3. Solutions of Metals

Soil was contaminated by aqueous solutions of metals. The metal ions selected for the experiment were Cu^2+^, Ni^2+^, and Zn^2+^, which are among the most widespread pollutants in the environment [49]. The following salts of the metals were used as sources of Cu^2+^, Ni^2+^, and Zn^2+^: CuSO_4_·5H_2_O, NiSO_4_·7H_2_O, ZnSO_4_·7H_2_O, and CuSO_4_·5H_2_O. Solutions of the metal salts were added to soil only once, on the day of setting up the experiment. Data on the solutions are given relative to cation forms of the metals, and not the total content of the salts. Soil with the sunflower but without the metals or sorbents served as a control. The dose of heavy metals applied to the soil was determined on the basis of the currently valid Ordinance of the Minister of the Environment of 1 September 2016 (Poland) on the method of assessing the pollution of the Earth’s surface [53].

### 2.2. Design of the Experiment

The experiment with *Helianthus annuus* L. was carried out under controlled conditions in a greenhouse at the University of Warmia and Mazury in Olsztyn (NE Poland). The phytoremediation plant selected for the trials was the sunflower, which is the fourth most common oil plant grown worldwide in terms of the cropped acreage [54,55]. According to the FAO data [56], the main sunflower producers are Ukraine, Russia, and Argentina. *Helianthus annuus* L. is highly tolerant to heavy metals [57]. Iram et al. [58] drew attention to the fact that sunflowers grown on polluted land can be used for alternative power production based on green chemistry methods.

In order to ensure the optimum supply of nutrients for the sunflower, soil in each pot was enriched with the basic elements, such as N, P, K, and Mg, before sowing the crop into pots. The whole experiment was divided into four parts: (1) unpolluted objects, (2) polluted with copper, (3) polluted with nickel, and (4) polluted with zinc. Six different sorbents were applied in order to reduce the negative effect of heavy metals on yields of the sunflower and on the microbiological and biochemical properties of soil. The layout of the greenhouse experiment is presented in Table 1. Before sunflowers were harvested from the pots, the leaf greenness index SPAD (Soil and Plan Analysis Development) was determined with a SPAD 502 Chlorophyll Meter 2900P. The measurements were made in eight replications. Next, the yields of the sunflower’s aerial parts and roots were determined.

### 2.3. Methods of Microbiological, Biochemical and Physicochemical Analyses of Soil

On the day of harvest, soil samples were collected, passed through a sieve with the mesh size 2 mm, and submitted to determinations of the basic microbiological and biochemical properties. The scope of microbiological analyses included determinations, in four replications. The microorganisms were isolated on the following media: Bunt and Roviry [59] for organotrophic bacteria, on the microbiological medium of Kuster and Williams (1971) with the addition of nystatin and an actidione according to Parkinson [60] for actinomycetes, and on Martin’s medium [61] for fungi. The soil material (10 g of soil) with sterile saline (90 cm^3^) was shaken on a laboratory shaker type 358A (Elpin, Mińsk Mazowiecki, Poland) for 30 min at a speed of 120 rpm. The 10^−5^ and 10^−6^ dilutions were used to determine the number of organotrophic bacteria and actinomycetes, while 10^−3^ and 10^−4^ dilutions were used for fungi. Petri dishes with all cultures were incubated in an incubator at 28 °C (PSelecta Incudigit, Barcelona, Spain). The number of colony-forming units (cfu) was determined using a colony counter with a magnifying glass. The specification of the microbial substrates used was previously described in the publication Zaborowska et al. [62]. The number of microorganisms served as a matrix to determine the colony development index (CD) and the ecophysiological index of the diversity of organotrophic bacteria, actinomycetes, and fungi [63]. The CD index of microorganisms was calculated from the following formula:

CD = [N1/1 + N2/2 + N3/3… N10/10] × 100,(1)

where N1, N2, N3, …, N10 denote the sum of the quotients of colony numbers of microorganisms identified in particular days of the study (1, 2, 3, …, 10) and the sum of all colonies in the entire study period.

The index of ecophysiological diversity (EP) was determined using the following formula:

EP = −Σ(pi × log10 pi),(2)

where pi is the quotient of the number of colonies of microorganisms from particular days of the study and the sum of all colonies from the entire study period.

The biochemical analyses consisted of determinations, in three replications, of the activity of seven enzymes: indicators of the C cycle (dehydrogenase, *β*-glucosidase, and catalase), N cycle (urease), P cycle (acid phosphatase and alkaline phosphatase), and S cycle (arylsulphatase).

The activity of dehydrogenases (µmol TFF·kg^−1^ d.m. soil·h^−1^) was measured by the release and detection of formazan (TFF) [64], of urease (mmol N-NH_4_·kg^−1^ d.m. soil·h^−1^) was measured as a function of ammonium ions, of *β*-glucosidase, acid phosphatase, alkaline phosphatase, and arylsulfatase (mmol PN·kg^−1^ d.m. soil·h^−1^) was a measured as a function of *p*-nitrophenol (PN) [65], and of catalase (mol O_2_·kg^−1^ d.m. soil·h^−1^) was measured as a function of oxygen [66]. The specific assay procedures for all enzymes (buffers, temperature and duration of incubation, and reaction stop) were described in detail in our previous publication [67]. The activities of dehydrogenases, *β*-glucosidase, urease, acid phosphatase, alkaline phosphatase, and arylsulfatase were determined colourimetrically using a Perkin-Elmer Lambda 25 spectrophotometer (Waltham, MA, USA), while that of catalase was determined by titration with potassium permanganate.

Before starting the research, the soil particle size composition was determined in three repetitions using the aerometric method [68,69], and the contents of total nitrogen (N_total_) [70], organic carbon (C_org_) [71], available P and K [72], Mg [73], and nickel, copper, and zinc in royal water extracts were determined by flame and electrothermal absorption spectrometry according to PN-ISO 11047 [74]. The pH was also determined in 1 mol KCl·dm^−3^ [75], in addition to the hydrolytic acidity (HAC) and sum of exchangeable base cations (EBC) [76]. On the basis of the HAC and EBC values, the cation exchange capacity (CEC) and alkaline cation saturation (ACS) were calculated. The abovementioned determinations were performed using standard methods described in our earlier work [77].

### 2.4. Data Analysis and Statistical Processing

The evaluation of the effect of heavy metals on microorganisms, soil enzymes, and the yield of *Helianthus annuus* L. was estimated on the basis of Equation (3), while the effect of sorbents was estimated using Equation (4).

(3) $${\mathrm{IF}}_{\mathrm{HM}}=\frac{A_{\mathrm{HM}}}{A}-1,$$

where IF_HM_ is the index of the influence of particular heavy metals on the number of microorganisms, enzyme activity, or yield of *Helianthus annuus* L., A_HM_ is the number of microorganisms, activity of enzymes, or yield of plants in soil contaminated with heavy metals, and A is the number of microorganisms, activity of enzymes, or yield of plants in uncontaminated soil.

(4) $${\mathrm{IF}}_{\mathrm{Ad}}=\frac{A_{\mathrm{Ad}}}{A}-1,$$

where IF_Ad_ is the index of the influence of individual sorbents on the number of microorganisms, enzyme activity, or yield of *Helianthus annuus* L., A_Ad_ is the number of microorganisms, enzyme activity, or the yield of plants in the soil with the addition of sorbents, and A is the number of microorganisms, the activity of enzymes, or the yield of plants in the soil without sorbents.

The research results were processed statistically in Statistica 13.3 [78]. A two-way analysis of variance (ANOVA) test was run in order to compare the means between the different sorbents and soil pollution with the heavy metals. Significant differences between the means were identified with the Tukey’s test (HSD). Statistical calculations were performed at *p* = 0.05. Moreover, in order to expose the relationships between the results, principal component analysis (PCA) and Pearson’s simple correlation were completed. In addition, the contribution of the analysed independent variables (η^2^) to the shaping of dependent variables was determined with ANOVA. Indices illustrating the impact of the heavy metals and sorbents on microorganisms, soil enzymes, and yield of the sunflower were presented on heat maps with a dendrogram of their similarities. This was performed in the programme Rstudio [79] using R v3.6.2 [80] and the gplots library [81].

## 3. Results

### 3.1. Microorganisms

All the tested heavy metals decreased the abundance of Org, Act, and Fun in soil, although in different degrees (Table 2). 

**Figure 1:**
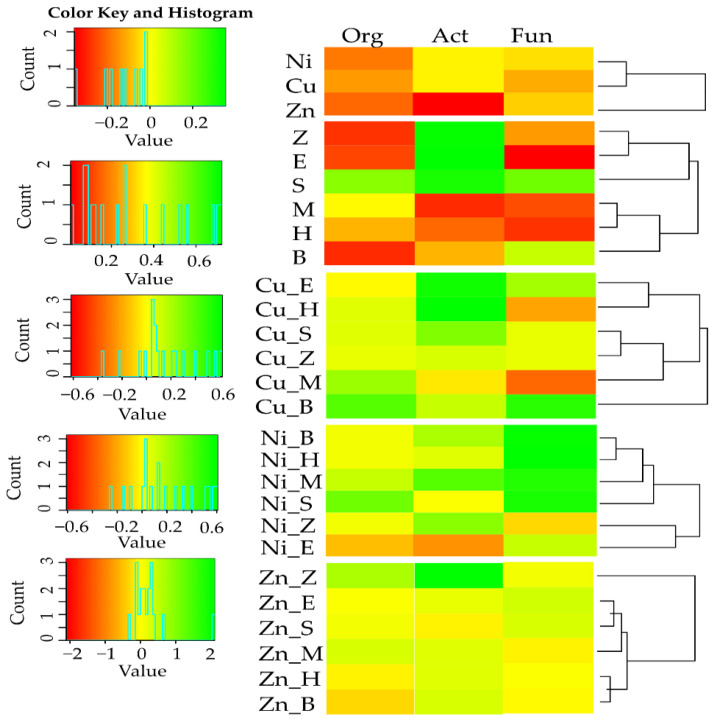
Index of the influence of heavy metals and sorbents on the number of microorganisms. Org—organotrophic bacteria; Act—actinomycetes; Fun—fungi; M—molecular sieve; H—halloysite; S—sepiolite; E—expanded clay; B—biochar; Z—zeolite; Cu—ion Cu^2+^; Ni—ion Ni^2+^; Zn—ion Zn^2+^.

In soil where no sorbents were used, the count of Org was from 13.7% (soil with Cu^2+^) to 20.5% (soil with Zn^2+^), of Act was from 2.1% (soil with Cu^2+^ and Ni^2+^) to 35.4% (soil with Zn^2+^), and of Fun was from 3.6% (soil with Ni^2+^) to 10.9% (soil with Cu^2+^), lower than in unpolluted soil. This was confirmed by values of the impact factor of heavy metals (IF_Hm_) on soil microorganisms (Figure 1). All the sorbents stimulated the multiplication of Org and Fun in the objects unpolluted with heavy metals, as well as the multiplication of Act, except zeolite. In the soil unpolluted with heavy metals, the effect of remediation substances was varied. The negative effect of Cu^2+^ on Org and Act was alleviated by all the analysed sorbents, while the effect on Fun was remediated by all substances except expanded clay. In the objects polluted with Ni^2+^, positive values of IF_Ad_ for all the groups of microorganisms appeared following the application of a molecular sieve, sepiolite, and expanded clay to the soil. Furthermore, halloysite and biochar limited the negative effect of Ni^2+^ on Org, while biochar and zeolite had the same influence on Act. The negative effect of Zn^2+^ on Org was mitigated by a molecular sieve, halloysite, sepiolite, and zeolite, while that on Act was mitigated by all remediation substances except expanded clay, and that on Fun was mitigated by all the sorbents except halloysite. An analysis of the IF_Ad_ values substantiated the conclusion that the interaction of most of the sorbents with the tested heavy metals stimulated the multiplication of soil microorganisms. In the soil contaminated with Ni^2+^, a molecular sieve proved to be the most effective remediation substance (a rise in the count of Org by 52.4%, of Act by 67.6%, and of Fun by 56.1%), while zeolite was most effectively moderated the effect of Zn^2+^ (an increase in the count of Org by 69.0%, of Act by 210.4%, and of Fun by 13.31%). Finally, the best conditions for the development of microorganisms in the soil contaminated with Cu^2+^ were created by a molecular sieve (for Org), halloysite (for Act), and expanded clay (for Fun).

**Figure 2:**
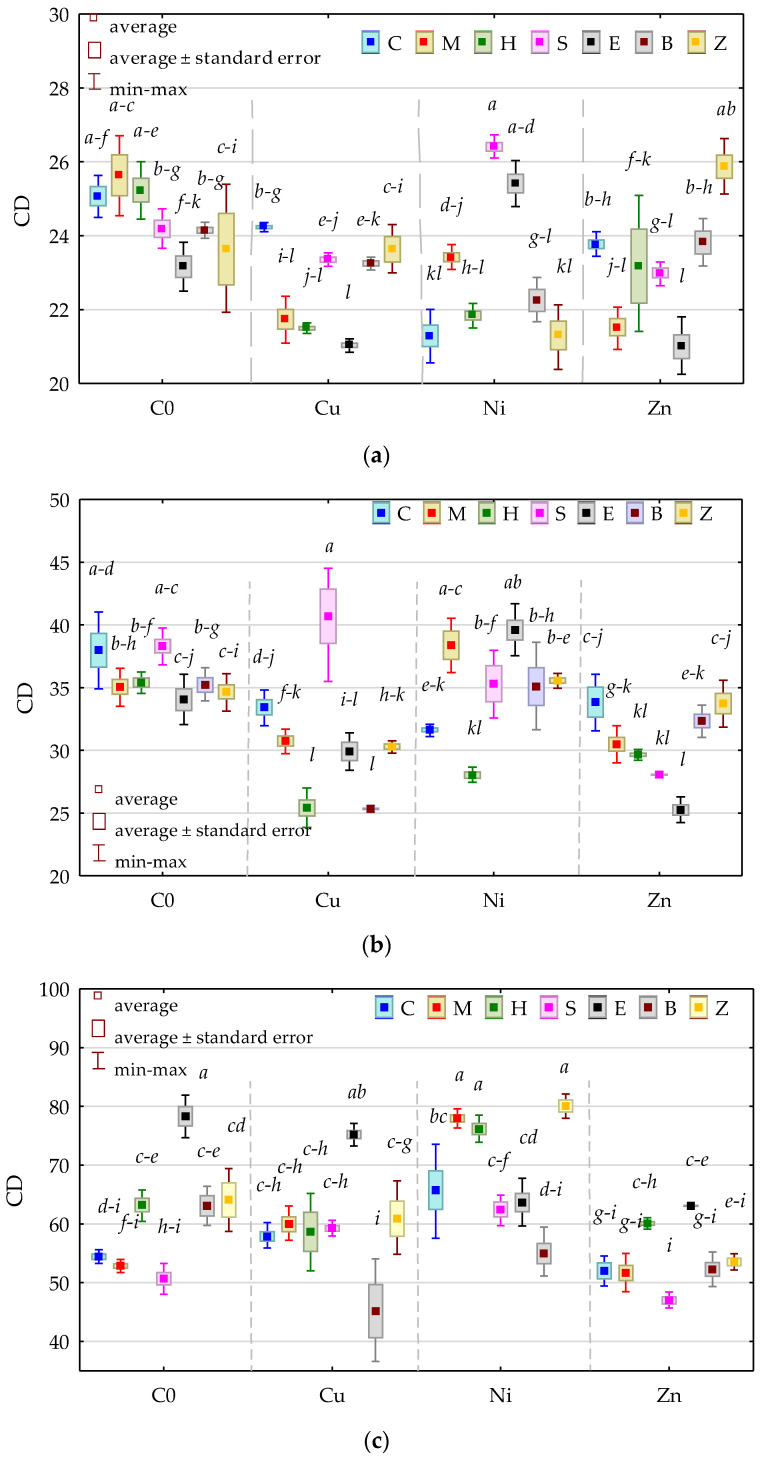
The colony development index (CD) (**a**) organotrophic bacteria, (**b**) actinomycetes, and (**c**) fungi. C—control; M—molecular sieve; H—halloysite; S—sepiolite; E—expanded clay; B—biochar; Z—zeolite; C0—uncontaminated soil; Cu—ion Cu^2+^; Ni—ion Ni^2+^; Zn—ion Zn^2+^. Homogeneous groups were calculated for CD values determined for all sorbents (denoted with letters a–i).

The colony development index (CD) for Org in the unpolluted objects without the sorbents was 37.97, compared to 25.07 for Act and 54.43 for fungi (Figure 2). The CD values for Org and Act isolated from the soil polluted with any of the heavy metals were lower than those from the objects not exposed to the pressure of the pollutants, while the CDs of Fun were higher in the soil with Cu^2+^ and Ni^2+^. The impact of the applied sorbents on the CD of microbial colonies was not unequivocal. The CD for the Fun colonies isolated from the soil void of heavy metals increased significantly only under the effect of expanded clay, while, in the case of Org and Act, it remained stable and ranged from 34.663 (zeolite) to 38.312 (sepiolite) for Org and from 23.637 (zeolite) to 25.634 (a molecular sieve).

The interaction of most sorbents with Cu^2+^ and Zn^2+^ lowered, although not always statistically significantly, the CD of Org and Act. The values of the CD for Org and Act isolated from the soil polluted with Ni^2+^ were shaped reversely. As for Fun, the application of expanded clay to the soil with Cu^2+^ and Zn^2+^ and the addition of a molecular sieve or halloysite to the soil exposed to the pressure of Ni^2+^ significantly increased the values of the CD of these microorganisms.

**Figure 3:**
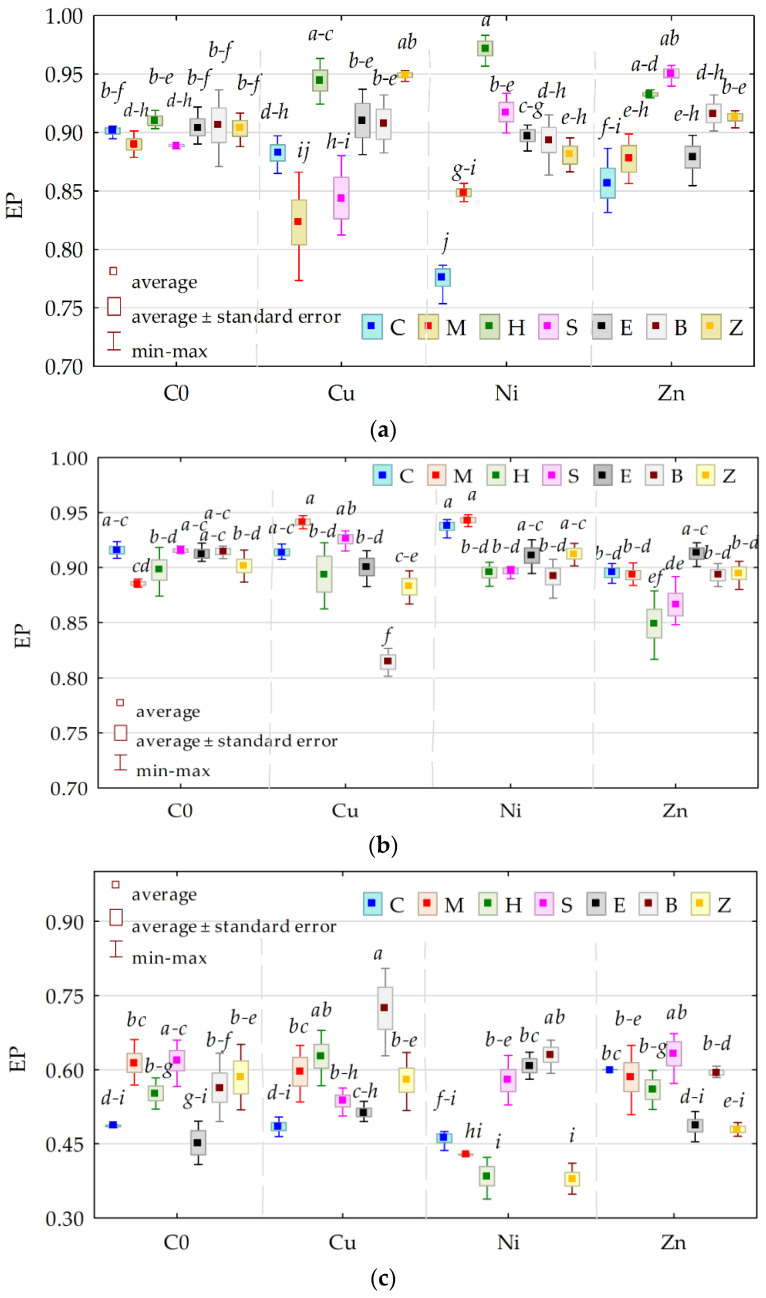
Ecophysiological diversity index (EP) (**a**) organotrophic bacteria, (**b**) actinomycetes, and (**c**) fungi. Homogeneous groups were calculated for EP values determined for all sorbents (denoted with letters a-i). The abbreviations are explained under Figure 2.

The ecophysiological diversity (EP) of Org decreased significantly under the influence of Ni^2+^, while that of Fun increased in the presence of Zn^2+^ in soil (Figure 3).

While the applied sorbents did not change the ecophysiological diversity of Org in the unpolluted soil, these substances significantly raised the EP value of Org in the soil polluted with Ni^2+^. A significant increase in the EP value in the soil contaminated with Cu^2+^ was only recorded after the application of two remediation substances, i.e., halloysite and zeolite, while, in the soil with Zn^2+^, it was recorded after the application of biochar and zeolite. The ecophysiological diversity of Act isolated from the soil submitted to the pressure of Cu^2+^, Ni^2+^, and Zn^2+^ and remediated was at an approximately identical level, regardless of the type of applied substance. However, it was only biochar that significantly decreased the EP value in objects with Cu^2+^ and Ni^2+^, halloysite in soil with Ni^2+^ and Zn^2+^, and sepiolite in soil with Ni^2+^, while expanded clay raised the EP in soil with Zn^2+^. The EP value of Fun isolated from the soil unpolluted with heavy metals was significantly increased by a molecular sieve and sepiolite. Regarding the soil polluted with Cu^2+^, a similar effect was observed in the soil amended with a molecular sieve, halloysite, and biochar, while, in the soil contaminated with Ni^2+^, it was observed after the application of sepiolite, expanded clay, and biochar. None of the remediation substances increased the ecophysiological diversity of Fun in the soil with Zn^2+^.

### 3.2. Soil Enzymes

The activity of all the analysed enzymes was inhibited by Cu^2+^, Ni^2+^, and Zn^2+^, which is evidenced by the results obtained from the particular objects (Table 3).

**Figure 4:**
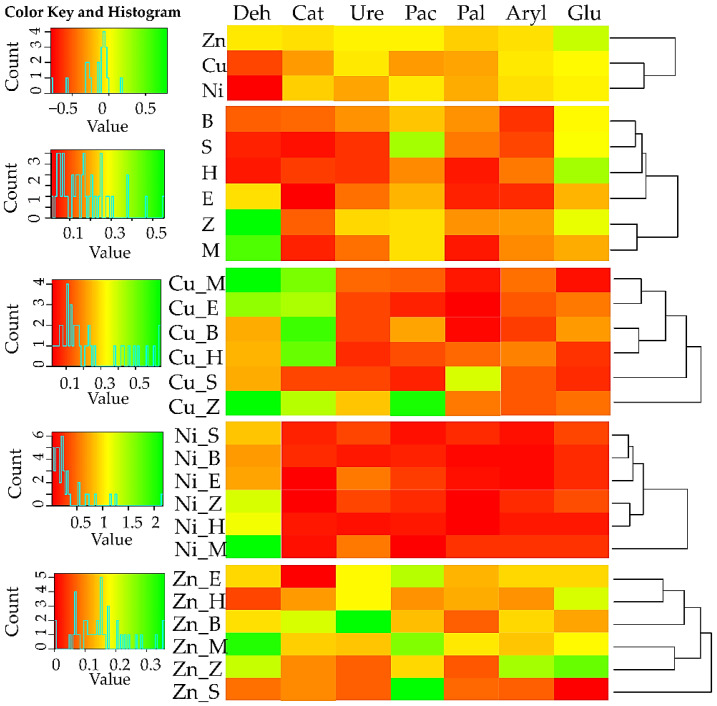
Index of the influence of heavy metals and sorbents on the activity of soil enzymes. The abbreviations are explained under Figure 1.

The enzymes Aryl and Glu turned out to be more resistant to the impact of the analysed heavy metals (Figure 4). Positive values of the IF_Ad_ prove that the applied sorbents stimulated the activity of all enzymes, although not to the same extent. The highest impact in this context was produced by zeolite, a molecular sieve, and biochar. Considering the average value of the IF_Ad_, irrespective of the type of tested enzymes, a molecular sieve and zeolite were the most effective remediation substances in the soil polluted with Cu^2+^ and Ni^2+^, while a molecular sieve and biochar were the most effective in the soil contaminated with Zn^2+^.

### 3.3. Yield

Of the tested heavy metals, only Ni^2+^ significantly disturbed the growth and development of *Helianthus annuus* L., which resulted in a decrease in the yield of aerial parts by 53.6% and roots by 45.7% (Table 4). The yield of aerial parts of *Helianthus annuus* L. in the objects unpolluted with heavy metals was significantly increased by the application of biochar and zeolite to the soil, whereas, in the soil contaminated with Cu^2+^, both sepiolite and biochar were effective remediation substances, and, in the soil contaminated with Ni^2+^, all the sorbents except expanded clay were effective. None of the substances significantly changed the aerial parts of the sunflower in the soil with Zn^2+^. In the control object and in the soil with Cu^2+^, the development of *Helianthus annuus* L. roots was significantly improved by halloysite and zeolite, in the soil with Ni^2+^, it was significantly improved by a molecular sieve, and, in the pots with Zn^2+^, it was significantly improved by a molecular sieve, sepiolite, and zeolite.

**Figure 5:**
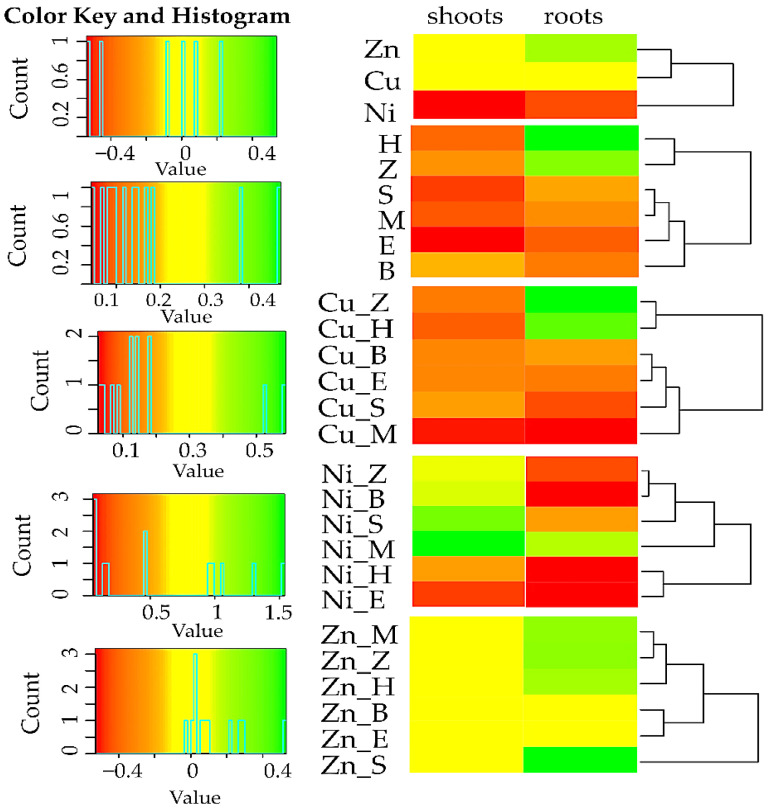
Index of the influence of sorbents on the d.m. yield aerial parts of *Helianthus annuus* L. The abbreviations are explained under Figure 1.

The above relationships were confirmed by the values of the impact of sorbents (IF_Ad_) on the yield of the test plant (Figure 5). Disturbances in the synthesis of chlorophyll were caused only by Ni^2+^, as confirmed by the lowest leaf greenness index (21.88) (Table 5). These undesirable changes were completely alleviated by the activity of a molecular sieve, sepiolite, biochar, and zeolite. The application of Cu^2+^ and Zn^2+^ to soil did not cause significant changes in the SPAD values.

### 3.4. Relationships between the Analysed Properties—PCA and Correlation Analyses

**Figure 6:**
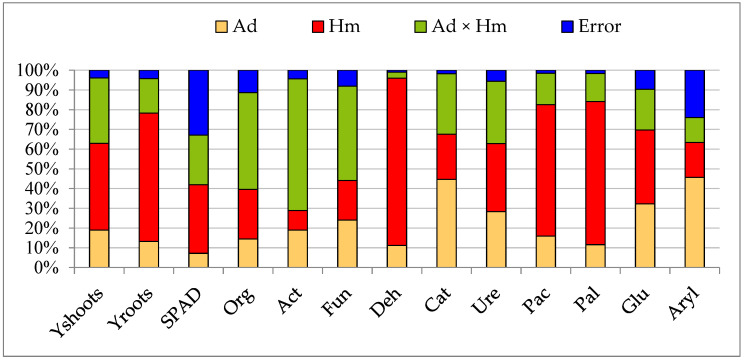
The contribution of independent variables (η^2^) in influencing the plant yield, SPAD index, the number of microorganisms, and the activity of soil enzymes. Ad—adsorbent; HM—heavy metals; Y_shoots_—yield of shoots; Y_roots_—yield of roots; Org—organotrophic bacteria; Act—actinomycetes; Fun—fungi; Deh—dehydrogenases; Cat—catalase; Pac—acid phosphatase; Pal—alkaline phosphatase; Glu—*β*-glucosidase; Aryl—arylsulphatase.

All the independent variables were affected both by the soil contamination with heavy metals (Hm) and by the application of the sorbents (Figure 6). The abundance of microorganisms was shaped by the soil contamination with heavy metals within the range of 9.84% (Act) to 25.16% (Org), and of soil enzymes within the range of 17.75% (Aryl) to 84.75% (Deh), while being affected by the remediation substances within the range of 14.45% (Org) to 24.03% (Fun) and within the range of 11.18% (Deh) to 45.66% (Aryl).

The count of Org and the activity of Deh, Ure, Pac, Pal, and Glu were determined to a higher degree by the heavy metals, while the counts of Act and Fun, as well as the activity of Cat and Aryl, were more strongly dependent on the sorbents. The yield of aerial parts of *Helianthus annuus* L. was determined at 43.98% and the yield of roots was determined at 65.07% by the heavy metals. In turn, the sorbents affected the two types of yield at 18.98% and 13.19%, respectively.

**Figure 7:**
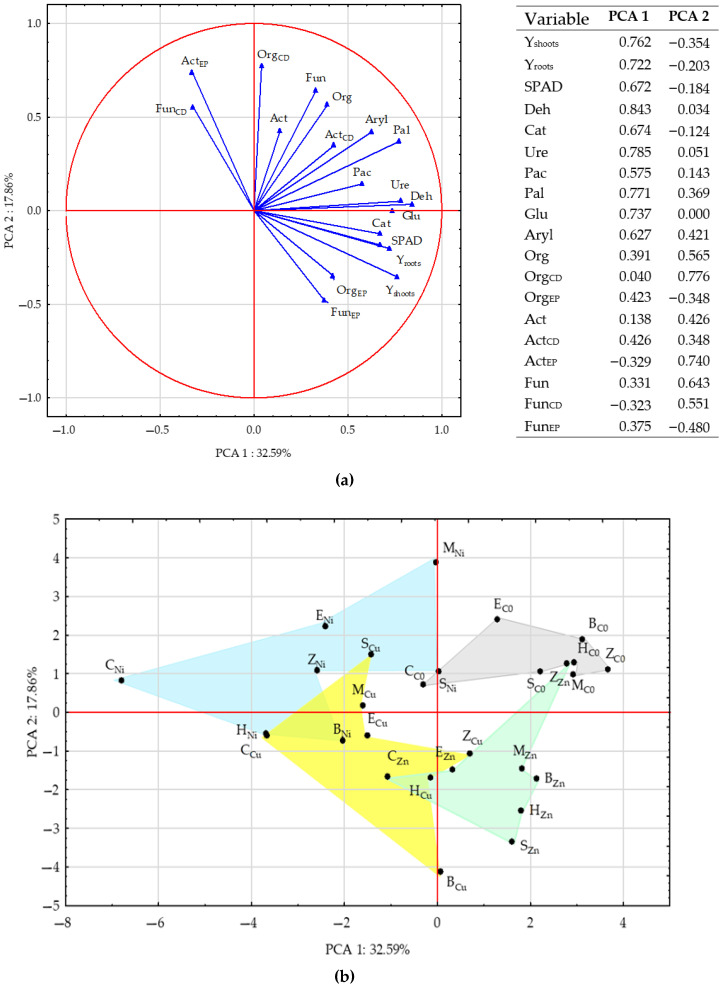
Test results presented by PCA: (**a**) figures representing primary variables; (**b**) cases.

**Figure 8:**
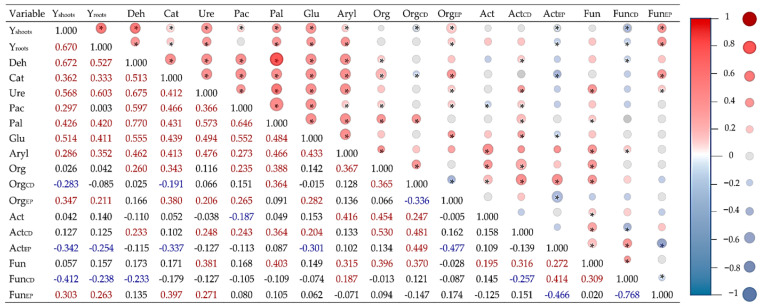
Pearson’s simple correlation coefficients, *n* = 112; Org—organotrophic bacteria; Act—actinomycetes; Fun—fungi; Deh—dehydrogenases; Cat—catalase; Pac—acid phosphatase; Pal—alkaline phosphatase; Glu—β-glucosidase; Aryl—arylsulphatase; Org—organotrophic bacteria; Act—actinomycetes; Fun—fungi; Y—yield of *Helianthus annuus* L.; CD—colony development index; EP—ecophysiological diversity index; * significant for *p* = 0.05.

The PCA (Figure 7) and the correlation analysis (Figure 8) and included all the data (all the sorbents and heavy metals). Among the enzymes, those whose activities were strongly correlated were Deh, Ure, Glu, and Cat. High correlation was also detected between the activities of Aryl and Pal, as well as between the SPAD indicator and the yielding of *Helianthus annuus* L. A negative correlation appeared between the indicators Fun_EP_ and Fun_CD_ versus Act_CD_, and between Org_CD_ and Org_EP_. The soil unpolluted with the heavy metals was positively affected by all the sorbents. The most severe turbulences were induced by nickel, followed by copper, while zinc was responsible for the weakest changes. The soil contaminated with nickel was most effectively remediated by sepiolite, while the soil exposed to the pressure of copper or zinc was most positively affected by zeolite.

## 4. Discussion

### 4.1. The Response of the Soil Microbiome to the Pressure of Cu^2+^, Ni^2+^, and Zn^2+^

Undoubtedly, consequences of the dispersion of Cu^2+^, Ni^2+^, and Zn^2+^ in the natural environment are linked predominantly to interference with the soil microbiome, which is perceived as a parameter rapidly responding even to small changes in the environmental stress [82]. In this experiment, all the tested heavy metals were powerful inhibitors of the abundance of organotrophic bacteria, actinomycetes, and fungi, which attests to the cytotoxic and genotoxic effects of these elements on the cellular level [83]. Importantly, heavy metals lead to excessive production of malondialdehyde (MDA) and are, therefore, responsible for peroxidation of lipids [84]. However, milder effects of the tested xenobiotics was expected, if only for the fact that numerous mechanisms have been identified among microorganisms that are responsible for detoxication. The finding that Ni^2+^ most severely disturbed the microbiological balance of the soil, thereby depressing the ecophysiological diversity of organotrophic bacteria, could be related to the fact that microorganisms activate the czcCBA gene-encoded efflux pumps and the cadA- and ZntA-induced ATPase efflux system towards Zn^2+^ [85] and CopA pumps towards Cu^2+^ that remove cytosolic copper in compilation with the ATOX1/Atx1 homologue [86,87], which is controlled by two transcription regulators, CueR and CsoR [88,89]. Moreover, the protein CutA1 binds Cu^2+^ using this process for the regulation of genes engaged in the tolerance to Cu^2+^ [90]. However, the toxicity of heavy metals should also be considered through the prism of the moderating effect of the soil pH. High pH induces a tendency to form insoluble metal carbonates and phosphates [91]. Importantly, at an elevated pH, the mobility and bioavailability of heavy metals in the soil also increases. This can be attributed to the different energy of input and output of metal cations to complexing organic ligands, which is closely related to dissolution/precipitation reactions and redox processes in soil [92,93]. As the soil pH decreases, the number of sorption sites for the cations Cu^2+^, Ni^2+^, and Zn^2+^ also decreases, which results in the greater mobility of these cations; hence, their negative impact escalates [94]. There is a definite pH value for each metal ion at which the extreme adsorption of those metal ions occurs. This also applies to the ionisation and dissociation of the sorbent molecule [95]. Admittedly, according to the CD values for organotrophic bacteria and actinomycetes, the applied xenobiotics changed the structure of these groups of microorganisms from r strategists to K strategists [96]. Nevertheless, both Ni^2+^ and Cu^2+^ raised the multiplication rate of fungi. This is probably because heat-shock proteins of fungi, such as HSP88 and HSP98, are engaged in the formation of copper nanominerals by fungi [97], or due to the production of copper oxalate crystals as a detoxifying by-product by a broad pool of mould fungi [98]. Liu et al. [97] also reported that the biomineralisation of Cu^2+^ with the participation of fungi is associated with gluconeogenesis because of the strong affinity of triose-phosphate isomerase to mycogenic copper nanoparticles. The tolerance of the fungi *Aspergillus awamori*, *Aspergillus flavus*, and *Aspergillus niger* to Ni^2+^ was also confirmed by Rose and Devi [99]. It is not without reason that a molecular sieve in our study was found to be effective in the remediation of soil polluted with Ni^2+^ and Cu^2+^, while zeolite was found to be effective in soil with Zn^2+^. Strong adsorption of metal ions by a molecular sieve is ensured by a large number of nanopores. This in turn is owed to the silica hydroxyl group dissociating into Si–O− and H^+^. The mechanism underlying this process is based on electrostatic interactions and ionic exchange on the Si surface [100]. In turn, zeolite is a microporous crystalline aluminosilicate with high thermal stability and chemical resistance correlated with the silicate–aluminium oxide ratio [101]. Boros-Lajszner et al. [52] noted improved biological parameters of soil contaminated with Ni^2+^, and Ismael [102] reported the same for soil polluted with Zn^2+^ after the application of zeolite, thus providing more evidence in favour of the current research results. In the research of Strachel et al. [51], biochar and molecular sieve proved to be effective minerals stimulating the proliferation of organotrophic, copiotrophic, oligotrophic, and actinomycetes in soil contaminated with Zn^2+^. As with halloysite, they did not induce an increase in the number of fungi, but nevertheless increased the ecophysiological diversity of this group of microorganisms. In turn, Boros-Lajszner et al. [103] indicated a molecular sieve and zeolite as sorbents positively influencing the number of organotrophic bacteria, actinomycetes, and fungi in soil under heavy-metal pressure. The authors concluded that the molecular sieve was more effective in improving the microbial activity of the soil than zeolite.

### 4.2. Activity of Soil Enzymes

The main responsibility of soil enzymes, constitutive or induced proteins, is to moderate the pace of reactions of organic matter decomposition and release of nutrients to the soil environment [104]. Potentially, however, any heavy metal or metalloid may disturb the biochemical balance of soil, depending on the dose of a xenobiotic and duration of the exposure [105]. The toxic influence of Cu^2+^, Ni^2+^, and Zn^2+^ on the activity of all the analysed soil enzymes arises from the denaturation of the enzyme’s protein, induced by the pressure of these metals, competition with metal ions essential in the formation of the enzyme–substrate complexes, or interactions with the active sites of enzymes [47]. Ure and Glu proved to be sensitive only to Cu^2+^ and Ni^2+^, while Deh, Cat, Pac, Pal, and Aryl responded negatively to all the xenobiotics. The hypothesis that the dehydrogenase enzymes are the most sensitive indicators of early soil degradation changes [106,107] has been supported by results of many studies [48,108,109,110]. Furthermore, the response of urease to Cu^2+^ can be attributed to the blocking of thiol groups by this metal, and binding with histidine residues in protein [111]. According to Mazzei et al. [112], nickel ions are most effective in the activation of urease, because every αβγ subunit of the protein of this enzyme possesses an active centre with two nickel ions. It could, therefore, be expected that the activity of urease should be stimulated after the contamination of soil with N^2+^. However, it needs to be emphasised that Ni^2+^ shows a negative effect on the activity of urease, same as C^2+^, mainly when present in the form of complexes, through the interaction not only with histidine nitrogen atoms, but also with oxygen atoms of glutamic acid residues of this enzyme’s amino acids [113]. The demonstrated weaker inhibitory power of the xenobiotic towards arylsulphatase and β-glucosidase corresponds with the trends determined by Strachel [51] in soil polluted with Zn^2+^ and by Boros-Lajszner et al. [52] in soil exposed to Ni^2+^. Nonetheless, Kandziora-Ciupa et al. [114] noted significant inhibition of the activity of *β*-glucosidase under the pressure of Zn^2+^ in both rhizosphere and non-rhizosphere soils. Hence, it is important to search for soil remediation techniques which will be economically viable and only negligibly interfering with the homeostasis of soil environments [115]. A scope of such technologies includes the application of biochar, which alleviated the adverse impact of Zn^2+^ on the biochemical activity of soil, and a molecular sieve and zeolite, which relieved the negative effect of the toxicity of Cu^2+^ and Ni^2+^. The effectiveness of biochar can be attributed to both its ability to elevate the soil pH, by degrading carbonates and hydroxides in this adsorbent [116], and to the fact that biochar can absorb heavy metals by reduction, conversion of pollutants to an organic form, electrostatic attraction, or complexation [38,117]. The effectiveness of zeolite can be associated with the affinity of this clay mineral to binding heavy metals and the high capability of adsorbing pollutants by forming surface complexes [118]. The ability to adsorb metal ions by reactive minerals is closely related to silica hydroxyl group, which can be dissociated into Si–O^−^ and H^+^, resulting in a negatively charged sorbent surface [119]. Furthermore, the surface of mineral-Si contains huge number of silanol groups (Si–OH). Due to their ability to ion exchange, the silanol groups strongly immobilised heavy metals in the soil. Moreover, in the research by Ma et al. [120], after the application of the clay mineral in the cultivated soil, the amount of organic matter decreased, suggesting that it favoured its decomposition and availability of organic matter and ultimately results in an increase in soil enzyme activity. Yang et al. [121] observed the stimulation of the activity of oxidoreductase, alkaline phosphatase, and urease, enzymes responsible for the circulation of carbon, phosphorus, and nitrogen in soil enriched with clay minerals.

### 4.3. Plant

Phytoremediation is one of the methods for the remediation of soils contaminated with heavy metals. Over 664 plant species are able to hyperaccumulate pollutants [39]. They include the sunflower (*Helianthus annuus* L.), which demonstrates high tolerance to heavy metals [50]. Thus, it was expected that the sunflower would show a positive response to the exposure to Cu^2+^, Ni^2+^, and Zn^2+^. However, it was found out that Ni^2+^, in contrast to Cu^2+^ and Zn^2+^, significantly distorted the growth and development of the sunflower’s yield. Chhotu et al. [122] observed that the exposure to low Ni^2+^ doses resulted in the stimulation of the lengthening of roots and stems of sunflowers, while higher amounts of this element limited the growth of the plant, mostly the elongation of roots and stems. Consequences of the toxicity of Ni^2+^ are manifested as the chlorosis of leaves, harmful effect on the thickness of mesophyll cells, size of vascular bundles, disturbances in mineral nutrition, and eventually, the inhibition of the growth of plants and depressed yields [123]. A very common response to the stress induced by metals, interestingly including Cu^2+^, consists of the synthesis of hydroxyl ion radicals in the Fenton and Haber–Weiss reactions, responsible for peroxidation of lipids [124,125]. A milder response of the sunflower to the negative effect of Cu^2+^ and Zn^2+^ might be attributed to the diversity of chelators of the sunflower roots. Flavonoids released by the plant roots can chelate both with Cu^2+^/Zn^2+^ and with Ni^2+^ [126], and Cu^2+^ and Zn^+^ can be bound by siderophores exerted by plants [127]. Moreover, production of metallothioneins, proteins engaged in detoxication and storage of metals, is induced mainly by Zn^2+^ and Cu^2+^ [128]. Noteworthy is the fact that Cu^2+^ plays a considerable role in photosynthesis, respiration, and protection from oxidative stress [129]. These reports align with our results, which lead to the conclusion that only Ni^2+^ significantly interfered with the synthesis of chlorophyll. According to Turan et al. [43], a weakened photosynthesis process is a typical effect of Ni^2+^ pressure. The disruption of photosynthesis is caused by the competitive replacement of Mg ions in chlorophyll by Ni^2+^ [130], or by the limited transport of electrons from pheophytin to plastoquinone Q [131]. When evaluating the usefulness of the applied adsorbents through the prism of the sunflower’s response to both the remediation factor and its compilation with the heavy metals, it was observed that the expected role in the objects polluted with Cu^2+^ was performed by sepiolite and biochar, whereas, in soil exposed to Ni^2+^, this was performed by all the sorbents except expanded clay. A study carried out by Quartacci et al. [124] confirmed the effectiveness of biochar in immobilising Cu and reducing its phytotoxicity by as much as 97%. Yang et al. [132] reported that biochar decreased the extractable concentration of Cy by 97%, while Shaaban et al. [133] concluded that biochar considerably reduced the toxicity of Ni. In turn, Abad-Valle et al. [134] demonstrated that sepiolite not only contributed to the metabolic regeneration of soil contaminated with Zn^2+^, but also decreased the concentration of this metal in plant shoots by up to 45%.

## 5. Conclusions

All the heavy metals disrupted the microbiological and biochemical equilibrium of soil, lowering the abundance of microorganisms and activity of enzymes. The most severe turbulences were caused by Ni^2+^, followed by Cu^2+^, while the mildest ones were due to Zn^2+^. Values of the CD index prove that the heavy metals changed the relationships between rapidly and slowly growing microorganisms, leading to a shift in the balance of Org and Act from an r strategy to K strategy, and of Fun from a K strategy to r strategy. The ecophysiological diversity (EP) of Org decreased significantly under the influence of Ni^2+^, and that of Fun increased under the effect of Zn^2+^. Despite the adverse influence of all the tested heavy metals on soil microorganisms and enzymes, only Ni^2+^ had a significantly negative effect on the growth and development of *Helianthus annuus* L. This element also caused disturbances in the process of chlorophyll synthesis. The adverse effects of the heavy metals on the soil microbiome and its activity were mollified by the applied sorbents. However, the effects produced by these substances were varied and dependent on the soil property submitted to analysis. In the soil to which a dose of 150 mg Cu^2+^·kg^−1^ of soil was added, a significant increase in the EP was caused by only two of the applied remediation substances, i.e., halloysite and zeolite, while in the soil polluted with Zn^2+^, this was caused by biochar and zeolite. In turn, regardless of the type of soil enzyme, the most effective remediation substance in soils polluted with Cu^2+^ and Ni^2+^ were a molecular sieve and zeolite, while a molecular sieve and biochar were most effective in soil with Zn^2+^. The application of sorbents contributed to the increased biomass of plants, both aerial parts and roots, which is important in terms of effective phytoextraction of these metals from soil. However, irrespective of the analysed soil property, sepiolite can be recommended for remediation of soil polluted with Ni^2+^, and zeolite can be recommended in soil contaminated with Cu^2+^ or Zn^2+^. These two sorbents were able to alleviate most effectively adverse changed caused by the tested heavy metals in the soil environment.

## Figures and Tables

**Table 1:** The design of the greenhouse experiment with the sunflower.

An experimental plant	*Helianthus annuus L.*: 7 seeds were sown in a pot; after emergence, 4 plants were left in the pot
Soil	Sandy loam: sand 0.05–2.0 mm—60.63%, silt 0.02–0.05 mm—35.99%, and clay < 0.002 mm—3.38%. 1.07 g N_tot_·kg^−1^ d.m., 14.69 g C_org_·kg^−1^ d.m., 166.72 mg P·kg^−1^ d.m., 171.31 mg K·kg^−1^ d.m., 443.21 mg Mg·kg^−1^ d.m., 4.20 mg Cu·kg^−1^ d.m., 20.31 mg Zn·kg^−1^ d.m., 8.40 mg Ni·kg^−1^ d.m., pH_KCl_—6.00, EBC—145.00 mmol (+)·kg^−1^ d.m., HAC—13.50 mmol (+)·kg^−1^ d.m., CEC—158.50 mmol (+)·kg^−1^ d.m., ACS—91.49%.
Mineral fertilisation	110 mg N·kg^−1^ d.m. of soil in form of CO(NH_2_)_2_, 45 mg P·kg^−1^ d.m. of soil in form of KH_2_PO_4_, 110 mg K·kg^−1^ d.m. of soil in form of KH_2_PO_4_ + KCl, 20 mg Mg·kg^−1^ d.m. of soil in form of MgSO_4_·7H_2_O
Soil contamination with heavy metals	150 mg Cu·kg^−1^ in form of CuSO_4_·5H_2_O, 150 mg Ni·kg^−1^ in form of NiSO_4_·7H_2_O, 150 mg Zn·kg^−1^ in form ZnSO_4_·7H_2_O
Applied sorbents	Molecular sieve, halloysite, sepiolite, biochar, expanded clay, zeolite Bio.Zeo.S.01; all sorbents were used in the amount of 10 g·kg^−1^ d.m. of soil
The duration of the experiment	Total: 40 days
Number repetitions	The vases were arranged in random, complete blocks on tables in the same vegetation hall, with three repetitions per treatment, which made a total of 84 vases
Conditions	Conditions in the vegetation hall (University of Warmia and Mazury in Olsztyn, Poland, June–July 2020): day length—approximately 16 h, night-time—approximately 8 h, average air temperature—approximately 18 °C, watering up to 60% m.w.c. deionised water

EBC—sum of exchangeable base cations, HAC—hydrolytic acidity, CEC—cation exchange capacity, ACS—alkaline cation saturation.

**Table 2:** The number of bacteria, cfu·kg^−1^ d.m. of soil.

Object	C0	Cu^2+^	Ni^2+^	Zn^2+^	Average
Organotrophic bacteria,10^9^					
Control	30.62 *^d–h^*	26.42 *^f–j^*	24.85 *^h–j^*	24.35 *^h–j^*	26.56 *^C^*
Molecular sieve	41.61 *^a^*	33.38 *^c–e^*	37.86 *^a–c^*	27.02 *^e–j^*	34.97 *^A^*
Halloysite	32.78 *^c–f^*	28.84 *^d–h^*	30.76 *^d–h^*	25.88 *^g–j^*	29.57 *^C^*
Sepiolite	33.03 *^c–f^*	26.42 *^f–j^*	34.76 *^b–d^*	25.59 *^g–j^*	29.95 *^C^*
Expanded clay	34.41 *^cd^*	24.21 *^h–j^*	33.38 *^c–e^*	20.61 *^jk^*	28.15 *^BC^*
Biochar	38.21 *^a–c^*	27.31 *^e–i^*	32.14 *^c–g^*	21.40 *^i–j^*	29.76 *^C^*
Zeolite	32.93 *^c–f^*	27.61 *^e–i^*	17.45 *^k^*	41.17 *^ab^*	29.79 *^C^*
Average	34.80 ^*I*^	27.74 *^II^*	30.17 *^II^*	26.57 *^III^*	
Actinomycetes,10^9^					
Control	16.17 *^h–l^*	15.83 *^i–l^*	15.83 *^i–l^*	10.45 *^no^*	14.57 *^E^*
Molecular sieve	17.35 *^f–k^*	18.39 *^e–j^*	26.52 *^b^*	17.99 *^e–j^*	20.07 *^B^*
Halloysite	20.41 *^d–f^*	27.02 *^b^*	15.09 *^j–l^*	16.91 *^g–k^*	19.86 *^BC^*
Sepiolite	20.85 *^de^*	24.75 *^bc^*	17.80 *^e–j^*	11.49 *^mn^*	18.72 *^CD^*
Expanded clay	22.53 *^cd^*	16.91 *^g–k^*	16.12 *^h–l^*	7.74 *^o^*	15.83 *^E^*
Biochar	19.28 *^d–h^*	20.21 *^d–g^*	19.28 *^d–h^*	13.16 *^l–n^*	17.98 *^D^*
Zeolite	14.10 *^k–m^*	18.59 *^e–i^*	20.85 *^de^*	32.44 *^a^*	21.49 *^A^*
Average	18.67 *^II^*	20.24 *^I^*	18.78 *^II^*	15.74 *^II^*	
Fungi,10^7^					
Control	6.75 *^e–g^*	6.01 *^f–h^*	6.51 *^f–h^*	6.31 *^f–h^*	6.40 *^E^*
Molecular sieve	7.59 *^d–f^*	6.56 *^f–h^*	10.16 *^ab^*	6.36 *^f–h^*	7.67 *^B^*
Halloysite	9.71 *^a–c^*	7.39 *^d–f^*	4.14 *^i^*	4.98 *^hi^*	6.56 *^CD^*
Sepiolite	7.10 *^ef^*	7.30 *^ef^*	9.86 *^a–c^*	6.66 *^fg^*	7.73 *^B^*
Expanded clay	10.30 *^ab^*	9.66 *^a–c^*	10.06 *^ab^*	7.15 *^ef^*	9.29 *^A^*
Biochar	10.60 *^a^*	5.42 *^g–i^*	6.01 *^f–h^*	6.66 *^fg^*	7.17 *^BC^*
Zeolite	8.97 *^b–d^*	8.28 *^c–e^*	6.06 *^f–h^*	7.15 *^ef^*	7.62 *^B^*
Average	8.72 *^I^*	7.23 *^II^*	7.54 *^II^*	6.47 *^III^*	

C0—uncontaminated soil, Cu—ion Cu^2+^, Ni—ion Ni^2+^, Zn—ion Zn^2+^. Homogeneous groups were calculated separately for each group of microorganisms (denoted with letters a—o), separately on average regardless of the metal used (denoted with letters A—E), or separately on average regardless of the adsorbents used (denoted with letters I—III).

**Table 3:** Enzymatic activity in soil (kg^−1^ d.m. of soil·h^−1^).

Object	C0	Cu^2+^	Ni^2+^	Zn^2+^	Average
Dehydrogenases, µmol TFF					
Control	6.231 *^c–e^*	2.632 *^hi^*	1.273 *^j^*	5.840 *^e^*	3.994 *^D^*
Molecular sieve	9.134 *^a^*	4.329 *^f^*	4.041 *^fg^*	7.776 *^b^*	6.320 *^A^*
Halloysite	6.502 *^c–e^*	3.260 *^gh^*	2.733	6.129 *^de^*	4.656 *^C^*
Sepiolite	6.564 *^c–e^*	3.243 *^gh^*	2.377 *^hi^*	6.316 *^c–e^*	4.625 *^C^*
Expanded clay	7.776 *^b^*	3.854 *^fg^*	2.207 *^i^*	6.740 *^cd^*	5.144 *^B^*
Biochar	6.927 *^b–d^*	3.243 *^gh^*	2.156 *^hij^*	6.774 *^cd^*	4.775 *^C^*
Zeolite	9.694 *^a^*	4.329 *^f^*	2.852 *^hi^*	7.097 *^bc^*	5.993 *^A^*
Average	7.547 *^I^*	3.556 *^III^*	2.520 *^IV^*	6.667 *^II^*	
Catalase, mol O_2_					
Control	0.338 *^e–g^*	0.233 *^l^*	0.289 *^j^*	0.308 *^i^*	0.292 *^E^*
Molecular sieve	0.357 *^b–d^*	0.349 *^c–f^*	0.319 *^hi^*	0.353 *^c–e^*	0.344 *^B^*
Halloysite	0.364 *^a–c^*	0.353 *^c–e^*	0.334 *^f–h^*	0.342 *^d–g^*	0.348 *^B^*
Sepiolite	0.349 *^c–f^*	0.259 *^k^*	0.342 *^d–g^*	0.338 *^e–g^*	0.322 *^D^*
Expanded clay	0.342 *^d–g^*	0.334 *^f–h^*	0.304 *^ij^*	0.308 *^i^*	0.322 *^D^*
Biochar	0.379 *^a^*	0.364 *^a–c^*	0.353 *^c–e^*	0.372 *^ab^*	0.367 *^A^*
Zeolite	0.375 *^a^*	0.330 *^gh^*	0.304 *^ij^*	0.338 *^e–g^*	0.337 *^C^*
Average	0.358 *^I^*	0.318 *^III^*	0.321 *^III^*	0.337 *^II^*	
Urease, mmol N-NH_4_					
Control	0.223 *^e–g^*	0.208 *^h^*	0.162 *^i^*	0.216 *^fg^*	0.202 *^C^*
Molecular sieve	0.254 *^b–d^*	0.239 *^c–f^*	0.246 *^c–e^*	0.246 *^c–e^*	0.246 *^A^*
Halloysite	0.239 *^c–f^*	0.223 *^e–g^*	0.177 *^i^*	0.254 *^b–d^*	0.223 *^B^*
Sepiolite	0.239 *^c–f^*	0.231 *^d–g^*	0.216 *^fg^*	0.231 *^d–g^*	0.229 *^B^*
Expanded clay	0.254 *^b–d^*	0.231 *^d–g^*	0.246 *^c–e^*	0.254 *^c–e^*	0.246 *^A^*
Biochar	0.262 *^bc^*	0.231 *^d–g^*	0.185 *^hi^*	0.293 *^a^*	0.243 *^A^*
Zeolite	0.277 *^ab^*	0.262 *^bc^*	0.216 *^fg^*	0.231 *^d–g^*	0.246 *^A^*
Average	0.250 *^I^*	0.232 *^II^*	0.207 *^III^*	0.246 *^I^*	
Acid phosphatase, mmol PN					
Control	1.275 *^i–k^*	0.872 *^o^*	1.174 *^kl^*	1.236^*jk*^	1.139 *^E^*
Molecular sieve	1.593 *^bc^*	0.989 *^mn^*	1.227 *^jk^*	1.563 *^bc^*	1.343 *^B^*
Halloysite	1.483 *^c–g^*	0.975 *^m–o^*	1.356 *^hi^*	1.369 *^g–i^*	1.296 *^D^*
Sepiolite	1.755 *^a^*	0.934 *^no^*	1.320 ^h–j^	1.679 *^ab^*	1.422 *^A^*
Expanded clay	1.533 *^cd^*	0.934 *^no^*	1.510 *^c–f^*	1.521 *^c–e^*	1.375 *^B^*
Biohar	1.567 *^bc^*	1.068 *^lm^*	1.406 *^e–h^*	1.403 *^f–h^*	1.361 *^B^*
Zeolite	1.599 *^bc^*	1.400 *^f–h^*	1.421 *^d–h^*	1.428 *^d–h^*	1.462 *^A^*
Average	1.544 *^I^*	1.025 *^IV^*	1.345 *^III^*	1.457 *^II^*	
Alkaline phosphatase, mmol PN					
Control	1.349 *^bc^*	0.970 *^l^*	1.014 *^j–l^*	1.152 *^fg^*	1.121 *^C^*
Molecular sieve	1.406 *^b^*	1.024 *^j–l^*	1.252 *^de^*	1.344 *^bc^*	1.256 *^B^*
Halloysite	1.404 *^b^*	1.118 *^g–i^*	1.032 *^i–l^*	1.293 *^c–e^*	1.212 *^D^*
Sepiolite	1.544 *^a^*	1.337 *^b–d^*	1.239 *^ef^*	1.239 *^ef^*	1.340 *^A^*
Expanded clay	1.424 *^b^*	0.988 *^kl^*	1.120 *^g–i^*	1.299 *^c–e^*	1.208 *^C^*
Biochar	1.574 *^a^*	1.008 *^j–l^*	1.089 *^g–j^*	1.232 *^ef^*	1.226 *^BC^*
Zeolite	1.578 *^a^*	1.131 *^gh^*	1.060 *^h–k^*	1.225 *^ef^*	1.249 *^B^*
Average	1.468 *^I^*	1.082 *^III^*	1.115 *^IV^*	1.255 *^II^*	
Arylosulphatase, mmol PN					
ControI	0.188 *^b–g^*	0.174 *^fg^*	0.170 *^g^*	0.170 *^g^*	0.176 *^D^*
Molecular sieve	0.218 *^ab^*	0.202 *^a–f^*	0.214 *^a–c^*	0.194 *^a–g^*	0.207 *^A^*
Halloysite	0.216 *^ab^*	0.206 *^a–e^*	0.192 *^a–g^*	0.188 *^b–g^*	0.200 *^AB^*
Sepiolite	0.204 *^a–f^*	0.196 *^a–g^*	0.188 *^b–g^*	0.182 *^d–g^*	0.192 *^C^*
Expanded clay	0.200 *^a–g^*	0.196 *^a–g^*	0.184 *^c–g^*	0.196 *^a–g^*	0.194 *^C^*
Biochar	0.202 *^a–f^*	0.192 *^a–g^*	0.180 *^e–g^*	0.196 *^a–g^*	0.192 *^C^*
Zeolite	0.206 *^a–e^*	0.196 *^a–g^*	0.210 *^a–e^*	0.212 *^a–d^*	0.210 *^A^*
Average	0.207 *^I^*	0.194 *^II^*	0.191 *^II^*	0.191 *^II^*	
*β*-glucosidase, mmol PN					
Control	0.550 *^g–i^*	0.543 *^h–i^*	0.523 *^i^*	0.640 *^d–g^*	0.564 *^C^*
Molecular sieve	0.661 *^c–f^*	0.565 *^g–i^*	0.661 *^c–f^*	0.751 *^a–c^*	0.660 *^B^*
Halloysite	0.761 *^ab^*	0.592 *^f–i^*	0.595 *^f–i^*	0.773 *^ab^*	0.680 *^B^*
Sepiolite	0.709 *^b–e^*	0.587 *^f–i^*	0.693 *^b–e^*	0.640 *^d–g^*	0.657 *^B^*
Expanded clay	0.665 *^c–f^*	0.635 *^d–h^*	0.626 *^e–h^*	0.739 *^a–c^*	0.666 *^B^*
Biochar	0.702 *^b–e^*	0.661 *^c–f^*	0.626 *^e–h^*	0.713 *^b–e^*	0.676 *^B^*
Zeolite	0.721 *^b–d^*	0.629 *^d–h^*	0.701*^b–e^*	0.820 *^a^*	0.718 *^A^*
Average	0.681 *^II^*	0.602 *^IV^*	0.632 *^III^*	0.725 *^I^*	

The abbreviations are explained under Table 1. Homogeneous groups were calculated separately for each enzyme (denoted with letters a–o), separately on average regardless of the metal used (denoted with letters A–E), or separately on average regardless of the adsorbents used (denoted with letters I–IV).

**Table 4:** The yield of *Helianthus annuus* L. (d.m. g·pot^−1^).

Object	C0	Cu^2+^	Ni^2+^	Zn^2+^	Average
Shoots					
Control	25.27 *^cd^*	25.29 *^b–d^*	11.72 *^f^*	27.52 *^a–c^*	22.45 *^C^*
Molecular sieve	27.26 *^a–c^*	26.41 *^a–d^*	29.76 *^a^*	28.31 *^a–c^*	27.93 *^A^*
Halloysite	27.66 *^a–c^*	27.46 *^a–c^*	17.23 *^e^*	29.29 *^a^*	25.41 *^B^*
Sepiolite	27.04 *^a–c^*	29.75 *^a^*	27.11 *^a–c^*	28.19 *^a–c^*	28.02 *^A^*
Expanded clay	26.36 *^a–d^*	28.82 *^a–c^*	13.35 *^f^*	27.81 *^a–c^*	24.08 *^B^*
Biochar	29.89 *^a^*	28.88 *^ab^*	23.21 *^d^*	29.71 *^a^*	27.92 *^A^*
Zeolite	29.06 *^a^*	28.49 *^a–c^*	22.99 *^d^*	26.91 *^a–c^*	26.86 *^A^*
Average	27.51 *^I^*	27.87 *^I^*	20.77 *^II^*	28.25 *^I^*	
Roots					
Control	5.18 *^h–j^*	4.72 *^i–j^*	2.81 *^l^*	6.32 *^c–h^*	4.76 *^B^*
Molecular sieve	5.89 *^e–i^*	4.84 *^ij^*	5.78 *^f–i^*	8.00 *^b^*	6.13 *^A^*
Halloysite	7.63 *^bc^*	7.22 *^b–e^*	2.98 *^l^*	7.70 *^bc^*	6.38 *^A^*
Sepiolite	6.06 *^d–i^*	5.02 *^h–j^*	4.13 *^j–l^*	9.66 *^a^*	6.22 *^A^*
Expanded clay	5.63 ^g–i^	5.31 *^h–j^*	3.03 *^l^*	6.44 *^c–h^*	5.10 *^B^*
Biochar	5.80 *^e–i^*	5.55 ^g–j^	3.01 *^l^*	6.89 *^b–g^*	5.31 *^B^*
Zeolite	7.15 *^b–f^*	7.49 *^b–c^*	3.30 *^kl^*	8.14 *^b^*	6.52 *^A^*
Average	6.19 *^II^*	5.74 *^III^*	3.58 *^IV^*	7.59 *^I^*	

The abbreviations are explained under Table 1. Homogeneous groups were calculated separately for shoots (denoted with letters a–f) and roots (denoted with letters a–l), separately on average regardless of the metal used (denoted with letters A–C), or separately on average regardless of the adsorbents used (denoted with letters I–IV).

**Table 5:** Greenness index (SPAD) of *Helianthus annuus* L.

Object	C0	Cu^2+^	Ni^2+^	Zn^2+^
Control	35.99 *^a–c^*	35.63 *^a–c^*	21.88 *^ef^*	34.14 *^a–c^*
Molecular sieve	34.71 *^a–c^*	33.88 *^a–c^*	33.15 *^b–d^*	35.56 *^a–c^*
Halloysite	38.64 *^ab^*	36.01 *^a–c^*	27.46 *^e^*	38.63 *^ab^*
Sepiolite	35.18 *^a–c^*	37.53 *^a–c^*	34.15 *^a–c^*	36.83 *^a–c^*
Expanded clay	35.24 *^a–c^*	36.95 *^a–c^*	21.34 *^f^*	36.76 *^a–c^*
Biochar	39.14 *^a^*	32.78 *^b–d^*	32.73 *^b–d^*	37.31 *^a–c^*
Zeolite	33.49 *^a–c^*	32.90 *^b–d^*	32.55 *^cd^*	38.21 *^a–c^*

The abbreviations are explained under Table 1. Homogeneous groups were calculated for SPAD (denoted with letters a–f).

## Data Availability

Data are available by contacting the authors.

## Abbreviations

M—molecular sieve; H—halloysite; S—sepiolite; E—expanded clay; B—biochar; Z—zeolite. Cu—ion Cu^2+^; Ni—ion Ni^2+^; Zn—ion Zn^2+^; SPAD—the leaf greenness index; Org—organotrophic bacteria; Act—actinomycetes; Fun—fungi; Deh—dehydrogenases; Cat—catalase; Ure—urease; Pac—acid phosphatase; Pal—alkaline phosphatase; Aryl—arylsulfatase; Glu—*β*-glucosidase; CD—colony development index; EP—ecophysiological diversity index; C_org_ –total organic carbon; N_total_—total nitrogen; HAC—hydrolytic acidity; CEC—cation exchange capacity; ACS—alkaline cation saturation.
